# Effects of Severe Hypoxia on Bone Marrow Mesenchymal Stem Cells Differentiation Potential

**DOI:** 10.1155/2013/232896

**Published:** 2013-09-04

**Authors:** Claudia Cicione, Emma Muiños-López, Tamara Hermida-Gómez, Isaac Fuentes-Boquete, Silvia Díaz-Prado, Francisco J. Blanco

**Affiliations:** ^1^Rheumatology Division, INIBIC Hospital Universitario A Coruña, C/As Xubias S/N, 15006 A Coruña, Spain; ^2^CIBER-BBN-Cellular Therapy Area, Hospital Universitario A Coruña, C/As Xubias S/N, 15006 A Coruña, Spain; ^3^Catedra Bioiberica-University of A Coruña, Hospital Universitario A Coruña, C/As Xubias S/N, 15006 A Coruña, Spain; ^4^Department of Medicine, INIBIC University of A Coruña, Campus de Oza S/N, 15006 A Coruña, Spain; ^5^Department of Medicine, University of Santiago de Compostela, A Coruña, Spain; ^6^Osteoarticular and Aging Research Laboratory, Hospital Universitario A Coruña, C/As Xubias S/N, 15006 A Coruña, Spain

## Abstract

*Background*. The interests in mesenchymal stem cells (MSCs) and their application in cell therapy have resulted in a better understanding of the basic biology of these cells. Recently hypoxia has been indicated as crucial for complete chondrogenesis. We aimed at analyzing bone marrow MSCs (BM-MSCs) differentiation capacity under normoxic and severe hypoxic culture conditions. *Methods*. MSCs were characterized by flow cytometry and differentiated towards adipocytes, osteoblasts, and chondrocytes under normoxic or severe hypoxic conditions. The differentiations were confirmed comparing each treated point with a control point made of cells grown in DMEM and fetal bovine serum (FBS). *Results*. BM-MSCs from the donors displayed only few phenotypical differences in surface antigens expressions. Analyzing marker genes expression levels of the treated cells compared to their control point for each lineage showed a good differentiation in normoxic conditions and the absence of this differentiation capacity in severe hypoxic cultures. *Conclusions*. In our experimental conditions, severe hypoxia affects the *in vitro* differentiation potential of BM-MSCs. Adipogenic, osteogenic, and chondrogenic differentiations are absent in severe hypoxic conditions. Our work underlines that severe hypoxia slows cell differentiation by means of molecular mechanisms since a decrease in the expression of adipocyte-, osteoblast-, and chondrocyte-specific genes was observed.

## 1. Introduction

Mesenchymal stem cells (MSCs) are multipotent cells that can be expanded *ex vivo* and induced, either *in vitro* or *in vivo*, to terminally differentiate into multiple lineages [1–5]. These cells are located in bone marrow (BM), around blood vessels, in fat, skin, muscle, and other tissues, and their presence contributes to the reparative capacity of these tissues. MSCs from different tissue sources can have biologic distinctions. In this way, MSCs derived from bone marrow show a higher potential for osteogenic differentiation [6], while MSCs of synovial origin show a greater tendency toward chondrogenic differentiation [7]. Moreover, under identical culture conditions of differentiation, MSCs isolated from the synovial membrane show more chondrogenic potential than those derived from bone marrow, periosteum, skeletal muscle, or adipose tissue [8].

The recent use of autologous or allogenic stem cells has been suggested as an alternative therapeutic approach for treatment of cartilage defects [9], with these cells representing a promising resource for different tissue engineering and cell-based therapies [10]. The interests in MSCs and their possible application in cell therapy have resulted in a better understanding of the basic biology of these cells. Clinical trials for diseases such as osteogenesis imperfecta, graft-versus-host disease, and myocardial infarction have shown some promise, demonstrating the safe use of both allogeneic and autologous cells [11–13]. In particular, in the last years, researchers focused on the effect of oxygen tension on MSCs and on differentiation. BM is one of the few organs that is maintained in the body in a hypoxic state [14]. Hypoxia plays an important role during development and cell differentiation [15]. The effect of hypoxic culture conditions was analyzed and showed to improve MSCs yield and reduce cells expansion time compared to the standard protocols [16]. Hypoxic conditions promote chondrogenic differentiation and enhance cartilage protein synthesis through the upregulation of Sox9, type II collagen, and aggrecan [17, 18]. Concerning osteogenesis and adipogenesis, the studies are controversial, probably due to different time exposures of cells to hypoxia. Both an improvement [19] and a decrease [20] of the differentiation capacity towards these lineages have been reported.

In this study, we confirmed that the cells used in our experiments were MSCs based on the combination of the three minimal criteria proposed by the International Society for Cellular Therapy in 2006 [21]. Furthermore, we analyzed adipogenic, osteogenic, and chondrogenic differentiation induced by commercial media both in normoxic and severe hypoxic conditions. The differentiations were confirmed by histologic, immunohistochemical and quantitative real-time PCR techniques (qPCR).

## 2. Materials and Methods

### 2.1. Isolation and Culture of MSCs

The BM samples used to isolate MSCs (BM-MSCs) were obtained from three patients (mean age: 64 years, range: 55–82 years) who underwent total hip replacement. The donors were not selected; the samples submitted were processed as they arrived at the laboratory. This study was approved by the institutional review board, and informed consent was obtained from all subjects in the study.

Isolated BM cells were cultured under standard culture condition (humidified atmosphere with 5% CO_2_), in Dulbecco's Modified Eagle's Medium (DMEM), 20% fetal bovine serum (FBS), and 1% penicillin and streptomycin (P/S) (all from Sigma-Aldrich, St. Louis, MO, USA) until 90% confluent. Preplating for 15 minutes in the first two passages eliminated any fibroblasts remaining in the culture [22].

The cells were cultured and expanded until the beginning of the differentiations experiments. At this point, when cells reached 90% of confluence, they were trypsinized, washed, and submitted both to phenotypical analysis and to differentiation experiments.

### 2.2. Phenotypic Characterization Using Flow Cytometry

At the third passage, after culture expansion, the cells were trypsinized, washed, and analyzed by flow cytometry. Briefly, the human BM-derived cells were harvested by trypsinization, washed, and centrifuged at 300 g for 8 minutes. The cells were counted prior to flow cytometry, and a total of 2 × 10^5^ were transferred to fluorescence-activated cell sorting (FACS) polypropylene tubes (NUNC, VWR International, Denmark). The antibodies listed in Supplementary Table 1 were used for these experiments (See Supplementary material available online at http://dx.doi.org/10.1155/2013/232896). Optimal amounts of monoclonal antibodies (mAbs) were determined and added to each tube for 40 minutes at 4°C in darkness. Most antibodies were conjugated with fluorescein isothiocyanate (FITC) or phycoerythrin (PE) and were characteristic for markers associated with mesenchymal and hematopoietic lineages. When necessary, cells were incubated for 30 minutes at 4°C in darkness with a secondary FITC-conjugated antibody to allow binding to the primary antibody. A control tube for each of the chromogens used contained equivalent amounts of isotype standards. A minimum of 25,000 cell events per assay were acquired on a FACsCalibur flow cytometer (BD Biosciences, Madrid, Spain). Data were analyzed using CellQuest Software (BD Biosciences), and the results were expressed as positive percentage.

### 2.3. Multipotential Characterization

At the time of flow cytometry, the cells were differentiated towards three different lineages (adipocyte, osteoblast, and chondrocyte). Differentiation experiments were carried out both in normoxic and severe hypoxic conditions. The term “normoxic conditions” is used to indicate the standard culture condition corresponding to humidified atmosphere with 21% O_2_. The term “severe hypoxic conditions” is used to indicate the culture condition corresponding to an atmosphere with 1% O_2_. The culture conditions used to induce each differentiation are described in the following paragraphs.

### 2.4. Adipogenic Differentiation

At the 3rd passage BM-MSCs were detached using trypsin-EDTA (Sigma-Aldrich, St. Louis, MO, USA), seeded at 1.5 × 10^5^ cells/cm^2^ in a chamber slide (BD Falcon, France), and cultured in growth medium until confluence. Adipogenesis was induced by culturing for three weeks in hMSC commercial adipogenic differentiation medium (Lonza, Biowhittaker, Belgium), following the manufacturer's instructions. Each differentiation point was compared to a control point corresponding to cells cultured for the same period of time in DMEM with 20% FBS. Differentiation was confirmed by staining techniques and gene expression quantification using real time PCR (qPCR).

### 2.5. Osteogenic Differentiation

BM-MSCs at the 3rd passage were detached using trypsin-EDTA, seeded at 1.5 × 10^5^ cells/cm^2^ in a chamber slide, and cultured in growth medium until confluence. Osteogenesis was induced by culture for three weeks in hMSC Commercial Osteogenic Differentiation Medium (Lonza, Biowhittaker, Belgium). This culture medium was changed every 3-4 days. Each differentiation point was compared with a control point that corresponded to cells cultured for the same period of time with DMEM and 20% FBS. Differentiation was assessed through histological and qPCR techniques. 

### 2.6. Chondrogenic Differentiation

Chondrogenesis was assessed using the micropellet formation (2.5 × 10^5^ cells) technique [23], with some modifications. BM-derived cells from the 3rd passage were detached using trypsin-EDTA and centrifuged at 300 g for 10 minutes. The resulting pellet was cultured in hMSC Commercial Chondrogenic Differentiation Medium (Lonza, Biowhittaker, Belgium) for 2 weeks. The culture medium was changed every 3-4 days. Each differentiation point was compared with cells cultured for the same period of time with DMEM and 20% FBS. After 14 days, cell aggregates were embedded in Tissue-Tek OCT compound (Sakura Finetek) and frozen. The presence of hyaline cartilage-characteristic molecules, such as type II collagen and proteoglycans, was detected by histological, immunohistochemical, and qPCR techniques as described below.

### 2.7. Histological Analyses

For adipogenesis evaluation, differentiation was confirmed by detection of cytoplasmic lipid droplets by oil red O staining after cell fixation in 4% paraformaldehyde.

For osteogenesis evaluation, differentiation was analyzed by Alizarin red staining after cell fixation in 4% paraformaldehyde, to assess the presence of calcium deposits.

For chondrogenesis evaluation, 4 *μ*m-thick frozen sections of aggregates were stained with hematoxylin and eosin (HE), Masson's trichrome (MT), toluidine blue (TB), alcian blue (AB), and safranin O (SaO) for proteoglycans and collagens.

### 2.8. Immunohistochemical Analyses

For chondrogenesis evaluation, 4 *μ*m thick frozen sections were incubated with primary antibodies to detect the presence of types I (Abcam, Cambridge, UK) and II (Neomarker, Barcelona, Spain) collagens, and with a polyclonal antibody to detect aggrecan C-20 (Santa Cruz Biotechnology, Heidelberg, Germany) (Supplementary Table 2). The peroxidase/DAB ChemMate DAKO EnVision detection kit (Dako, Barcelona, Spain) was used to determine antigen-antibody interactions. Negative staining controls were achieved by omitting the primary monoclonal antibody. Samples were visualized using an optical microscope.

### 2.9. RNA Extraction

Isolation of total RNA from cell cultures was accomplished using trizol reagent (Invitrogen, Barcelona, Spain), following the manufacturer's protocol. RNA was assessed for quantity at 260 nm using a NanoDrop spectrophotometer (Thermo Scientific, Madrid, Spain). The A260/A280 ratio was calculated to assess quality and purity. For each sample, 1 *μ*g of total RNA was further processed in RT-PCR or stored at −80°C until used.

### 2.10. cDNA Synthesis

Before reverse transcription, the total RNA underwent DNase digestion (Fermentas, Spain) for complete removal of DNA contamination. Subsequently, the reverse transcription reaction was performed from 1 *μ*g of total RNA using SuperScript First-Strand Synthesis System for RT-PCR (Invitrogen, Spain), following the manufacturer's instructions. Briefly, 1 *μ*g of total RNA, 0.5 *μ*g oligo d(T), 0.5 mM of dNTP mix, and 3 *μ*L of DEPC-treated water were denatured at 65°C for 5 minutes and chilled on ice for at least 1 minute. Then, 2 *μ*L of 10x RT buffer, 5 mM MgCl_2_, 0.01 M DTT, and 40 U of RNaseOUT Recombinant Ribonuclease Inhibitor were mixed, collected by centrifugation, and incubated at 42°C for 2 minutes. After incubation, 50 U of SuperScript RT was added and incubated at 42°C for 50 minutes and 70°C for 15 minutes in a Thermocycler (Gene Amp PCR System 9700, Applied Biosystems, Spain). Finally, samples were chilled on ice and incubated with 2 U of RNAse H for 20 minutes at 37°C before proceeding to the next step.

Samples were stored at −20°C before the amplification of target cDNAs. Positive and negative controls were included in each experiment. RNA extraction, reverse transcription-PCR assay setup, and postreverse transcription-PCR product analysis were carried out in separate dedicated rooms to prevent crosscontamination.

### 2.11. Quantitative Real-Time Reverse Transcription-PCR Analysis (qRT-PCR)

Real-time PCR analysis was performed using the primers shown in Table 1 on a LightCycler 480 Instrument (Roche, Mannheim, Germany).

The PCR reaction consisted of 10 *μ*L of Master Mix 2x concentrate, 0.25 *μ*M of each forward and reverse primer, the cDNA template, and PCR-grade water up to a final volume of 20 *μ*L in the LightCycler 480 Multiwell Plate 96. The multiwell plate was loaded in the LightCycler 480 Instrument until the PCR program started.

The initial enzyme activation at 95°C for 10 minutes was followed by 50 cycles of target amplification consisting of three sequential steps: 95°C for 10 seconds (s), 61°C for 5 s, and 72°C 7 s. After amplification, a melting curve analysis was performed and a final cooling step was applied at 40°C for 20 s.

The single amplification and expected size of each PCR product were verified. The use of 2% agarose gel electrophoresis, stained with *SYBR Safe DNA gel stain *(Invitrogen), of all PCR products revealed a single band that corresponded to the single-amplified products as predicted by the PCR melting curve analysis.

PCR primers were positioned to span exon-intron boundaries, reducing the risk of detecting genomic DNA. The primers were purchased from Roche (Mannheim, Germany). The TATA box binding protein gene (TBP) was used as the internal control housekeeping gene to normalize the amount of target cDNA.

Data analysis was performed using LightCycler 480 Relative Quantification software (Roche). Relative levels of expression were calculated by the 2^−ΔΔCt^ method [24]. Each assay was done at least in triplicate and included marker-positive and marker-negative controls and reagent with no template controls. Each data was normalized against the housekeeping gene and compared with its corresponding control point of cells grown in DMEM with 20% FBS. For each gene expression, we assigned the value 1 to the lowest level of expression, and the other values were measured as relative expression levels (REL).

### 2.12. DNA Sequencing Analysis

At least one PCR product coming from each real-time PCR experiment was used as template DNA in order to verify the specificity of the amplified amplicon. PCR products were purified by an enzymatic method (ExoSAP-IT, Amersham Biosciences, Spain). DNA sequencing was performed on an ABI 3130XL (Applied Biosystems, Spain) sequencer using BigDye Terminators (Applied Biosystems, Spain). Forward and reverse specific primers used were the same employed for the qPCR experiments (Table 1).

### 2.13. Other Procedures

The procedures used to manipulate the nucleic acids were those described in *Molecular Cloning: A Laboratory Manual* by Sambrook et al. [25].

### 2.14. Statistical Analysis

Each experiment was repeated at least three times. The statistical significance of the differences between mean values was determined using a two-tailed *t*-test; *P* < 0.05 was considered significant. Results are expressed as the mean ± standard deviation (mean ± SD). 

## 3. Results

### 3.1. Isolation of BM-MSCs Populations

Spindle-shaped bipolar cells attached to the flask were observed at the first medium change at 48 hours of culture. We expanded, differentiated, and analyzed the surface antigens expression and multipotentiality of the cells isolated from three donors.

### 3.2. Characterization of Culture Expanded BM-MSCs

#### 3.2.1. Phenotypic Analysis

At the third passage, at the beginning of the differentiation experiments, the cells were characterized using flow cytometry. The antibodies used were selected to characterize BM-MSCs population. Briefly, we used CD34 and CD45, markers of hematopoietic stem/progenitor cells; SSEA-4 and Stro-1, markers of embryonic stem cells; CD90, CD73, CD105, CD29, CD44, CD106, and CD166, markers of mesenchymal stem/stromal cells.

**Figure 1:**
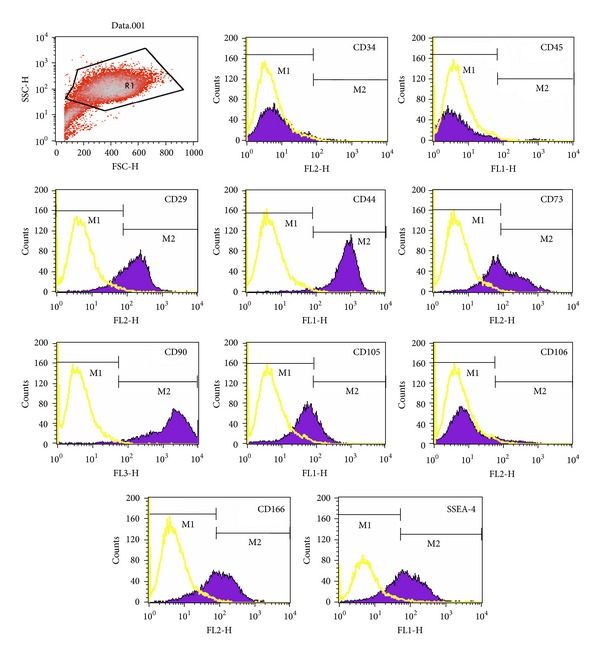
Phenotype of cells, at the third passage, isolated from human bone marrow of one donor. Antibodies listed in Supplementary Table 1 were used for this procedure. The figure shows representative histograms of multipotential mesenchymal stem cells obtained from FACS analysis. Black line signifies the specific antibody; yellow line represents the isotype control.

Figure 1 shows surface marker expressions of one donor. The cells from the three donors showed very similar expressions of surface markers. Each antigen expression value was expressed as positive percentage. In this way, CD73 and CD44 antigens were coexpressed at 94%. CD105 was expressed at 61% when alone and coexpressed with CD106 at 24%. On the other hand, CD166 was expressed at 92%. The percentages of CD90, CD29, and SSEA-4 expressions were 95%, 94%, and 83%, respectively. Cells from the three donors were negative for Stro-1, CD45, and CD34 expressions.

#### 3.2.2. Adipogenic Differentiation

**Figure 2:**
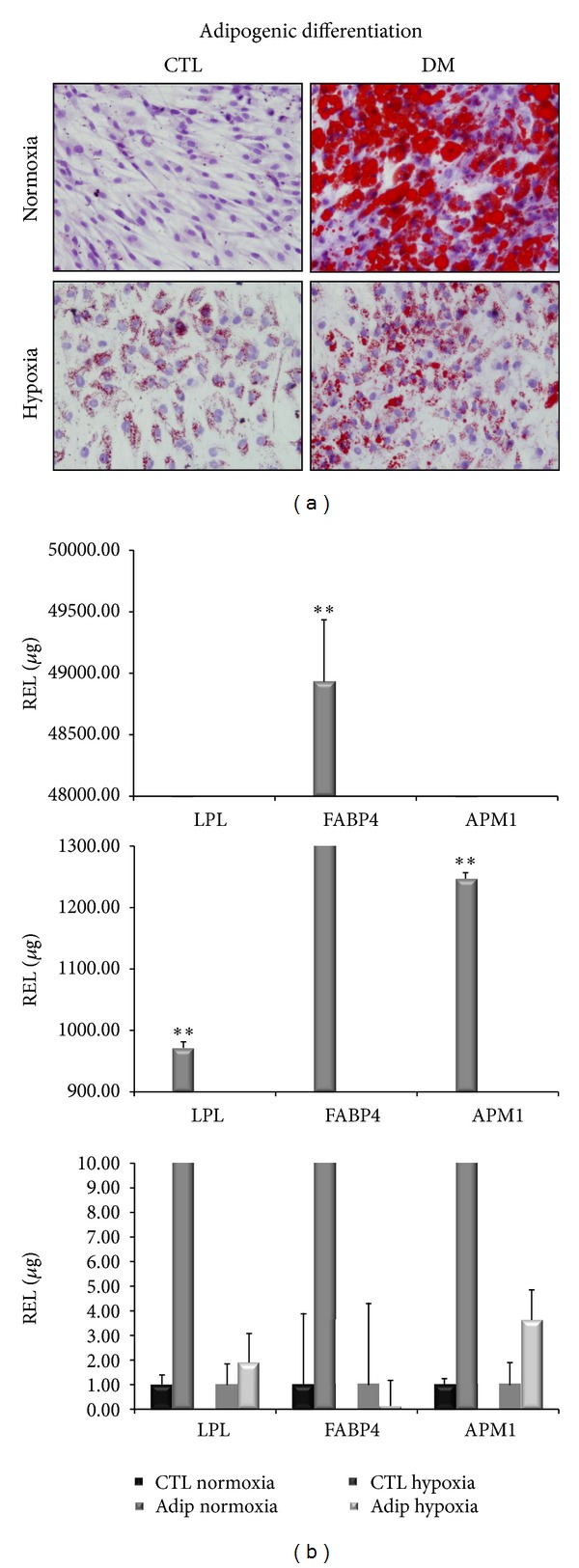
Staining techniques of adipogenic differentiated cells and adipogenic-specific gene expression levels in normoxic and hypoxic conditions. (a) Oil red O staining of the cells differentiated (DM) compared to their control point (CTL). The photos are relative to day 21 of cultures in normoxic and hypoxic conditions. (b) We compared CTL and treated cells (Adip.). Expression levels of LPL, FABP4, and APM1 at day 21 of cultures in normoxic (**P < 0.005) and hypoxic conditions.

After 21 days of culture with the appropriate differentiation medium or with 20% FBS for the control points, the cells were stained with Oil Red O for lipid droplets to evaluate adipogenic differentiation (Figure 2(a)). All the controls resulted negative for staining, showing the absence of lipid droplets. The cells grown in differentiation medium in normoxic conditions were positively stained, indicating differentiation, while the cells grown in differentiation medium in severe hypoxic conditions were not stained, indicating a marked decrease in differentiation.

To confirm these results, we extracted total RNA from cells treated in the same way, retrotranscribed 1 *μ*g of total RNA, and analyzed it through qPCR (Figure 2(b)). We measured the expression levels of LPL, FABP4, and APM1 marker genes for adipogenic differentiation. In each experiment, each gene expression level of cells treated with the differentiation medium was compared to cells cultured in DMEM with 20% FBS (control points). In normoxic conditions, treated cells showed higher levels of LPL, FABP4, and APM1 expressions compared to the control points (972.13, 48934.74, and 1247.03 times, resp.) and were statistically significant (*P* < 0.005). In severe hypoxic conditions, treatment of cells with differentiation medium was not able to induce differentiation, and gene expression levels of LPL, FABP4, and APM1 were 1.92, 0.14, and 3.64 times the control points. These results confirmed the histological stainings.

#### 3.2.3. Osteogenic Differentiation

**Figure 3:**
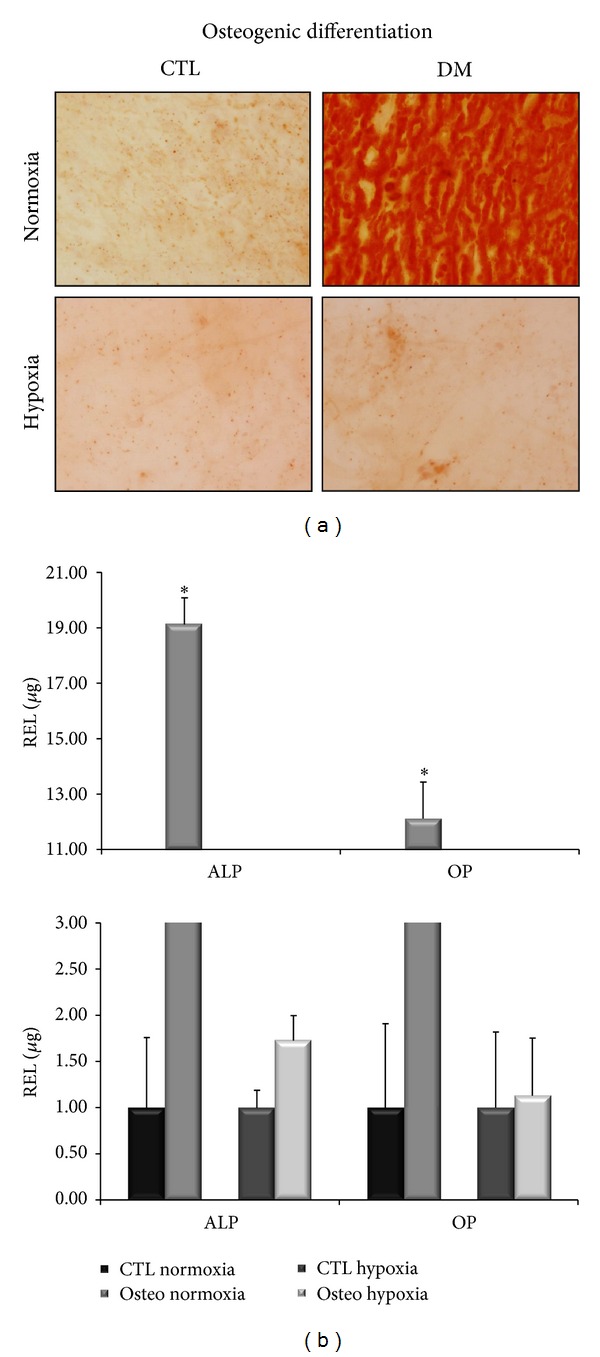
Staining techniques of osteogenic differentiated cells and osteogenic-specific gene expression levels in normoxic and hypoxic conditions. (a) Alizarin red staining of the cells differentiated (DM) compared to their control point (CTL). The photos are relative to day 21 of cultures in normoxic and hypoxic conditions. (b) We compared controls points (CTL) and treated cells (Osteo.). Expression levels of ALP and OP at day 21 of cultures in normoxic (*P < 0.05) and hypoxic conditions.

After 21 days of culture with the differentiation medium or with 20% FBS for the controls, the cells were stained with Alizarin Red for calcium deposits (Figure 3(a)). The controls were negative for staining. The cells grown in differentiation medium in normoxic conditions were positively stained, showing the presence of calcium deposits and the presence of differentiation, while the cells grown in differentiation medium in severe hypoxic conditions were not stained, indicating a marked decrease in differentiation.

To confirm these results, we extracted total RNA from cells differentiated under the same conditions, retrotranscribed 1 *μ*g of total RNA, and analyzed it through qPCR (Figure 3(b)). In each experiment, each gene expression level of cells treated with the appropriate differentiation medium was compared to cells cultured in DMEM with 20% FBS (control points). We measured the expression levels of ALP and OP genes for osteogenic differentiation. The three populations were capable of showing specific genes of osteoblast differentiation. In normoxic conditions, ALP and OP expression levels were, respectively, 19.14 times and 12.13 times higher than the control points, and the differences were statistically significant (*P* < 0.05), while in severe hypoxic conditions the expression levels of ALP and OP were, respectively, 1.73 and 1.13 times the controls.

#### 3.2.4. Chondrogenic Differentiation

**Figure 4:**
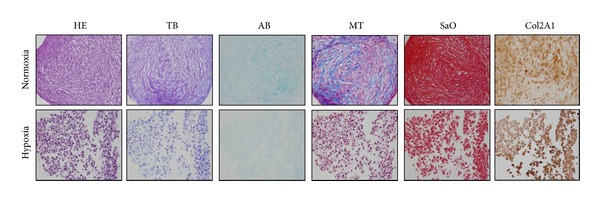
Histochemical (HE: hematoxylin-eosin; TB: toluidine blue; AB: alcian blue; MT: Masson's trichrome; SaO: safranin O) and immunohistochemical results for collagen type 2 (Col2A1) cells differentiated toward chondrocytes. The photos are relative to day 14 of cultures in chondrogenic differentiation medium in normoxic conditions (up) and in hypoxic conditions (down).

After 14 days of culture with the differentiation medium or with 20% FBS for the controls, the aggregates were analyzed by histochemistry using hematoxylin eosin (HE), Masson's trichrome (MT), toluidine blue (TB), alcian blue (AB), and Safranin O (SaO) staining and immunohistochemistry for aggrecan and type I and II collagens. These techniques confirmed chondrogenic differentiation in normoxic conditions and the absence of differentiation in severe hypoxic conditions. As shown in Figure 4, the presence of collagens and proteoglycans could be seen only in normoxic atmosphere. Immunohistochemistry results for aggrecan (Agg) and Col1 were negative (data not shown).

**Figure 5:**
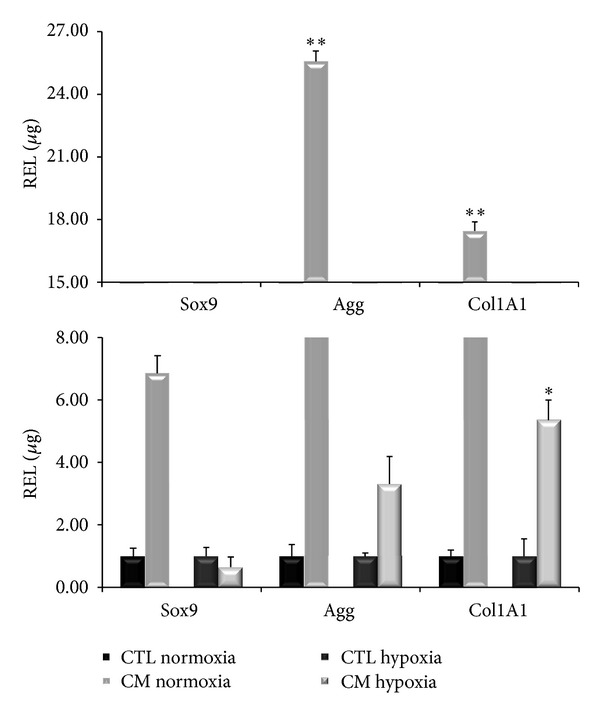
Chondrogenic-specific gene expression levels in normoxic and hypoxic conditions. In these figures we compared control points (CTL) and treated cells (CM). Expression levels of Sox9, Agg, and Col1A1 at day 14 of cultures in normoxic conditions (**P < 0.005) and in hypoxic conditions (*P < 0.05).

To confirm these results, we extracted total RNA from cells differentiated under the same conditions, retrotranscribed 1 *μ*g of total RNA, and analyzed it through qPCR (Figure 5). We measured the expression levels of Sox9, Agg, Col1A1, and Col2A1 marker genes for chondrogenic differentiation. In each experiment, each gene expression level of cells treated with the differentiation medium was compared to that of cells cultured in DMEM with 20% FBS (control points). In normoxic conditions (Figure 5), treated cells showed higher levels of Sox9, Agg, and Col1A1 expressions compared to the control points (6.87, 25.58, and 17.47 times, resp.) and were statistically significant (*P* < 0.005). In severe hypoxic conditions (Figure 5), the expression of Sox9, Agg and Col1A1 genes were 0.65, 3.31, and 5.36 times the control points. The Col2A1 gene was detected only in the treated cells in normoxic conditions with a Cp value of 31 (media of the three donors). Also these results confirmed the staining.

## 4. Discussion

Stem cells represent an attractive source for tissue engineering and reparative medicine [13]. Very little is known regarding the mechanisms that regulate stemness, self-renewal, and maturation of MSCs. The importance of tissue environmental factors, such as oxygen tension, in the induction of MSC differentiation has been previously studied [26–28]. Moreover, the studies from Simon and Keith [29] revealed that oxygen is a regulator of the stem cell biology.

Conventional *in vitro* cell cultures are often carried out under ambient oxygen concentration corresponding to 21% (defined as “normoxic”). However, physiologic oxygen pressure is lower and varies from tissue to tissue between 1% and 13% [30]. Therefore the 21% oxygen tension used as the normoxic condition exceeds the partial pressure of oxygen in most mammalian tissues, indicating that oxygen concentration during standard *in vitro* culture of primary human cells is often not adapted to the *in vivo* situation [31]. Marrow stem cells are generally cultured in the presence of 21% O_2_, but the pO_2_ in the bone marrow is much lower, between 1% and 7% [32]. This suggests for bone marrow-derived cells the use of hypoxic *in vitro* conditions resembling their natural physiological environment. Mathematical models of the pO_2_ distribution in human bone marrow suggest a gradient across the marrow from the relatively well-oxygenated sinuses to the rather hypoxic endosteal region [33]. It is well known that high pO_2_ could be toxic, causing oxidative stress, due to the generation of reactive oxygen species (ROS) that can damage DNA, proteins, and lipids [34]. Therefore excess electrons are transferred to oxygen leading to the harmful oxidation of adjacent molecules [35]. For this reason hypoxia may decrease the intracellular ROS production and accumulation leading to an increase in the metabolic efficiency [34]. In this way, cultivation of MSCs under hypoxic conditions mimics the natural microenvironment of these cells and represents an important prerequisite to study cell proliferation, differentiation, senescence, metabolic balance, and other physiological processes [34]. Moreover, the fact of studying BM-MSCs differentiation in the presence of low oxygen concentration may be relevant to understanding the lack of repair processes that occur with severe hypoxia such as in situations of severe vascular ischemia, which occurs in patients with atherosclerosis or diabetes and in patients with inflammatory conditions such as arthritis. 

Attending the low O_2_ tension of BM *in vivo*, we studied the capacity of BM-MSCs to differentiate toward adipocyte, osteoblast, and chondrocyte comparing severe hypoxic and normoxic conditions. To drive their differentiation, we treated cells isolated from three different donors with commercial medium. In all the experiments performed, in normoxic conditions cells from the three donors were able to differentiate toward adipogenic, chondrogenic, and osteogenic lineages. However, when the differentiation treatments were carried out under severe hypoxic conditions, the same cells from the same donors were not able to differentiate. Severe hypoxic environment inhibited completely the three-lineage differentiation potential of our BM-MSCs. The absence of differentiation capacity under hypoxia was seen both with histological and immunohistochemical analyses. In addition, qPCR analysis of the marker genes for each lineage confirmed these results. We saw an upregulation of the genes analyzed when the differentiations were carried out under normoxia and a loss of this induction under severe hypoxia. The way by which severe hypoxia slows cell differentiation is by means of molecular mechanisms; indeed we have seen a decrease in the expression of adipocyte-, osteoblast-, and chondrocyte-specific genes. Moreover, our MSCs were isolated from osteoarthritic (OA) patients. Therefore, this inhibition of cell differentiation may explain the lack of repair processes that occur with severe hypoxia in patients with inflammatory conditions such as osteoarthritis. Moreover, it is possible that MSCs from OA donors show different *in vitro* differentiation potentials than those isolated from normal donors. In this way, not only the influence of different oxygen tensions but also the origin of the isolated BM-MSCs may influence the *in vitro* differentiation capacity.

In the last years, several papers have focused on the effects of hypoxia on stem cells isolated from different tissues and different species [36–38]. The effects of low oxygen concentration on cell growth have also been examined by several authors in different cell models such as rat marrow-derived mesenchymal stem cells and murine fibroblast, respectively [39, 40]. In this regard, D'Ippolito et al. [41] demonstrated that low oxygen concentration (1, 3, 5, and 10% O_2_) increases cell proliferation, obtaining the maximal difference in cells maintained at 3% O_2_. Moreover, low pO_2_ inhibits osteoblastic differentiation, suggesting that low oxygen tension (pO_2_) seems to be a cell culture condition that is critical to maintain MSCs in a less differentiated state. They showed that lineage-specific differentiation of stem cells may be enhanced when these cells are exposed to higher pO_2_, indicating that oxygen concentration regulates the balance between self-renewal and differentiation of MSCs. In other works, Holzwarth et al. [31] assessed that reduced oxygen tension (1 to 3% O_2_) severely impaired adipogenic and osteogenic differentiations of human MSCs. Also, Malladi et al. [42] demonstrated that osteogenesis and chondrogenesis of adipose-derived mesenchymal cells were inhibited *in vitro* by hypoxic culture conditions (2% O_2_). All these data are in agreement with the “niche” model proposed by Schofield [43], where stem cells or more developmentally primitive cells in the BM are located within a specific area characterized by a unique microenvironment, low in pO_2_, that contributes to maintaining a limitless potential of stem cells to self-renew, that is, proliferate without differentiation. The same positive effect of low pO_2_ on maintaining stemness has also been shown in other stem cell types [44, 45].

In contrast, other groups reported positive effects of reduced oxygen tensions on MSCs plasticity [20, 46]. For example, Lennon et al. [39] reported that rat marrow stromal cells showed increased extracellular mineralization in cultures maintained at low pO_2_ compared to normoxic conditions. Also, in several *in vitro* studies, low oxygen concentrations have been found to stimulate differentiation processes, inducing the cells toward the osteogenic, adipogenic, or chondrogenic lineage [47, 48]. In this regard, Wang et al. [49] reported that adipose-derived mesenchymal cells cultured in 5% O_2_ and suspended in alginate beads showed a decrease in growth and an increase in chondrogenic differentiation. Moreover, Robins et al. [27] have shown that low pO_2_ can promote chondrocytic differentiation from mesenchymal stem cells. These contradictory data can be explained by the variety of species involved, the type of culture design (which would change the type of oxygen gradients to which the cells were exposed), differences of culture conditions (such as growth factor supplementation of the media), the method of differentiation applied, the pO_2_ chosen, and the time points studied. In this way, Grayson et al. [19] reported an increase in adipogenic and osteogenic differentiations in long-term 3D cultures of bone marrow-derived mesenchymal stem cells under hypoxia. Compared to our work and our results, in Grayson's experiments [19] cells isolated from bone marrow were both expanded and differentiated in 3D architecture under hypoxic conditions. In our work we applied severe hypoxic conditions only during the differentiation experiments, and the 3D culture system was only applied during chondrogenic differentiations. 

As known from the literature, several molecular pathways involved in cellular metabolism are altered under hypoxia [50]. MSCs are characterized by metabolic flexibility and are able to survive hypoxic conditions [34, 51]. Hypoxia stabilized hypoxia-inducible factor-1 *α* (HIF-1*α*). HIF-1*α* is a transcriptional factor that activates more than 40 genes and plays essential roles in a variety of cellular and systemic homeostatic responses to hypoxia [50]. This transcriptional factor downregulates mitochondrial oxygen consumption [52] and upregulates key markers of stem cells like Oct-4, Rex-1, and SSEA4 [41]. We hypothesized that the relationship between HIF-1*α*, cells proliferation, and stem cell markers could explicate our results and the absence of differentiation under severe hypoxic conditions. In addition, several papers support this hypothesis [16, 53, 54]. In this regard, Carrancio et al. [16] reported that hypoxic culture conditions compared to the standard protocols showed to improve MSCs yield and reduce cells expansion time. Also, Ohnishi et al. [54] analyzed the gene expression of rat BM-MSCs in hypoxia and observed an upregulation of genes involved in development, cell proliferation, and cell survival. Finally, Dos Santos et al. [53] reported that hypoxia promotes cell proliferation and expansion. During proliferation, the majority of the cells undergo cell division and cannot begin the cellular mechanisms involved in differentiation processes. Therefore the use of low pO_2_ levels to increase the *in vitro* survival or self-renewal of human mesenchymal stem cells represents an important improvement in stem cell research that could be crucial for obtaining a high number of cells for therapeutic purposes.

In conclusion, we have shown that severe hypoxic conditions are able to abolish the differentiation capacity of BM-MSCs when applied during *in vitro* directed differentiation. Our work underlines that severe hypoxia slows cell differentiation by means of molecular mechanisms since a decrease in the expression of adipocyte-, osteoblast-, and chondrocyte-specific genes was observed.

## Supplementary Material

Supplementary Table 1: Antibodies used for phenotypical characterization by flow cytometry.
Supplementary Table 2: Antibodies used for immunohistochemical analyses.

## Figures and Tables

**Table 1:** Sequences of qPCR primers used for the amplification of human mRNA corresponding to adipogenic, osteogenic, and chondrogenic-specific genes.

Gene name	Forward primer (5′-3′)	Reverse primer (5′-3′)	mRNA ID number
Sox9	gtacccgcacttgcacaac	tcgctctcgttcagaagtctc	NM_000346
Col2A1	gtgtcagggccaggatgt	tcccagtgtcacagacacagat	NM_001844
Aggrecan (Agg)	gcctacgaagcaggctatga	gcacgccataggtcctga	BC036445
Col1	gtgatgctggtcctgttggt	caccatcgtgagccttctct	NM_000088
FABP4	ggatgataaactggtggtgga	cacagaatgttgtagagttcaatgc	NM_001442
APM1	ggtgagaaaggagatccaggt	tgctgagcggtatacataggc	NM_004797
LPL	agaacatcccattcactctgc	ccatttgagcttcaacatgagt	NM_000237
ALP	gacggacccgtcactctc	gtgcccgtggtcaattct	NM_000478
OP	cgcagacctgacatccagt	ggctgtcccaatcagaagg	NM_000582
TBP	gcccatagtgatctttgcagt	cgctggaactcgtctcacta	NM_003194
