# Supplementary material for: Effects of Severe Hypoxia on Bone Marrow Mesenchymal Stem Cells Differentiation Potential
Source: Stem Cells Int. 2013 Sep 4;2013:232896. doi: 10.1155/2013/232896 (PMC3777136; doi:10.1155/2013/232896)
Supplement: Supplementary file 1 — Supplementary Table 1: Antibodies used for phenotypical characterization by flow cytometry. Supplementary Table 2: Antibodies used for immunohistochemical analyses. [file 232896.f1.zip › Supplemetary Material Table 2.pdf]

Supplementary table 2: Antibodies used for immunohistochemical analyses.

Specificity	Clon	Source
**Type I collagen**	COL-1	Abcam
**Type II collagen**	COLL-II	Chemicon
**Aggrecan C-20**	Polyclonal	Santa Cruz Biotechnology
