# Supplementary material for: Effects of Severe Hypoxia on Bone Marrow Mesenchymal Stem Cells Differentiation Potential
Source: Stem Cells Int. 2013 Sep 4;2013:232896. doi: 10.1155/2013/232896 (PMC3777136; doi:10.1155/2013/232896)
Supplement: Supplementary file 1 — Supplementary Table 1: Antibodies used for phenotypical characterization by flow cytometry. Supplementary Table 2: Antibodies used for immunohistochemical analyses. [file 232896.f1.zip › Supplementary Material Table 1.pdf]

Supplementary table 1: Antibodies used for phenotypical characterization by flow cytometry.

**Antibody**	**Specificity**	**Source**
**R-PE-CD29**	β1 Integrin	***BD Pharmingen***
**R-PE-CD34**	Hemopoietic progenitor cell antigen 1 (HPCA1)	***BD Pharmingen***
**FITC-CD44**	HCAM	***BD Pharmingen***
**FITC-CD45**	Leukocyte common antigen (LCA)	***BD Pharmingen***
**PE-CD73**	Ecto-5´-nucleotidase	***BD Pharmingen***
**PECy5-CD90**	Thy-1	***BD Pharmingen***
**FITC-CD105**	Endoglin, SH2	***Chemicon***
**PE-CD106**	VCAM-1	***AbD Serotec***
**R-PE-CD166**	ALCAM	***BD Pharmingen***
**SSEA-4**	Stage-specific embryonic antigen 4	**R&D *Systems***
**STRO-1**	Stromal antigen 1	***Developmental Studies Hybridoma Bank***
